# Conditionally Reprogrammed Cells as Preclinical Model for Rare Cancers

**DOI:** 10.3390/cancers17172834

**Published:** 2025-08-29

**Authors:** Ewa Krawczyk

**Affiliations:** Department of Pathology, Center for Cell Reprogramming, Georgetown University Medical Center, Washington, DC 20057, USA; ewa.krawczyk@georgetown.edu

**Keywords:** conditional cell reprogramming, rare cancers, cell culture, preclinical models, disease models in vitro

## Abstract

Despite their disadvantages, preclinical models in vitro are still crucial for every area of biomedical science. They remain a necessary basis for biological, biochemical, and mechanistic studies of pathophysiology of human disease, evaluation of diagnostic tests, assessment of vaccines, as well as screening of potential and repurposed drugs before they are adapted to clinical use. In contrast to animal models in vivo, preclinical in vitro models are cost and time effective. They are easier to use, and, in most cases, they are not associated with ethical concerns. Therefore, they are extensively used in cancer research. Conditional cell reprogramming (CCR) has been one of the novel technologies utilized as a preclinical model in vitro for various common cancers and other diseases. It may be even more important for the research related to rare cancers—elusive, difficult to study, and with insufficient number of relevant models available. Applications of this technology for the basic and translational studies of rare cancers are described in this article. Evaluation of the mechanisms of tumorigenicity and metastasis in neuroblastoma, neuroendocrine cervical carcinoma, ependymoma and astrocytoma, as well as screening of potential drugs and other therapeutic approaches for the laryngeal and hypopharyngeal carcinoma and adenoid cystic carcinoma, demonstrate that the CCR technology is a potential reliable model for various aspects of rare cancer research in the future.

## Simple Summary

Testing new and potential drugs before they are used in patients is an essential part of biomedical science. Assessing their safety and efficiency as well as researching the mechanisms of the disease—including cancer—is performed in preclinical disease models. Advancements in designing new models are especially important for rare cancers: they are difficult to study because of their uncommon occurrence. Conditional cell reprogramming, a relatively new and promising method of culturing cells in vitro, has been already shown useful for modeling various diseases. This article describes the basics of conditionally reprogrammed technology and its applications in rare cancer research.

## 1. Introduction

Preclinical disease models are indispensable in every field of biomedical sciences, including cancer research. They are utilized worldwide for testing possible drugs, vaccines, and diagnostic tests before they are used in the clinic, among others. However, preclinical models, especially preclinical models in vitro, are not perfect and they never precisely recapitulate the pathology and pathophysiology of human organisms. Therefore, there is a constant and urgent need to develop new, advanced, and more accurate preclinical disease models [1,2,3,4,5,6,7,8].

The most basic pre-clinical models are two-dimensional (2D) established cell lines [9]. These conventional cell lines are commercially available, can be cultivated in laboratories at a relatively low cost, are easy to use, and usually no ethical concerns (in contrast to the problems associated with experimental animals use [10]) are associated with them (with the notable exception of human embryonal cell lines [11]). Conventional 2D cell lines have been crucial for innumerable inventions in the biomedical field, from assessment of the efficacy and toxicity of new and repurposed drugs, to the study of genetic and biochemical mechanisms of numerous diseases, including various cancers [12,13,14]. Two-dimensional cells enable high-throughput research [15]: the researchers can obtain very large numbers of cells within relatively short periods of time; the cells can be then biobanked [16]; they are widely available at low cost; and their use does not require particularly high experience from the researchers. However, conventional established cell lines suffer from several weaknesses. They are normally used as monolayers in two-dimensional settings; hence, they do not represent the structure of the tissue (or the cancer) they were collected from [17,18]. Regarding the cell type, they are homogenous. Therefore, studies researching the interactions between different cell types within the tissue or the tumor are not possible [19]. Two-dimensional cell lines do not recapitulate the interconnection and interactivity of tumor microenvironment [20] and do not correctly reflect tumor complexity [21]. Importantly, cell lines kept in the laboratories for many passages accumulate various mutations, as well as karyotype and phenotype changes, and they do not resemble (genetically, physiologically, metabolically, etc.) the original cell line/tissue they were collected from anymore [6,22,23,24,25]. There is also limited number of control cell lines, since normal (tumor-corresponding) cells cannot be grown in laboratory settings indefinitely [26]. The biomedical research based on 2D cell lines is further jeopardized by cross-contaminations, misidentification, and mislabeling of cells used in laboratories on a daily basis [27,28,29,30,31,32], and it has to be emphasized that cell lines should be frequently authenticated to make sure that the experiments are performed using the appropriate models.

Nevertheless, conventional cell lines remain important for various areas of cancer research. Especially for common cancers, there is a variety of available cells representing many different types and different cancer stages, even in different populations of patients. In contrast to common cancers though, rare cancers are not very well-represented in the repositories of conventional cell lines [33]. By definition, rare cancers occur infrequently, are unique, and in numerous cases, there is no adequate number of preclinical models of them available to researchers. Thus, designing and developing new disease models, including two-dimensional in vitro models, becomes crucial for rare and unique malignancies [34,35,36,37].

## 2. Conditional Cell Reprogramming

One of these relatively new preclinical in vitro models is the conditional cell reprogramming (CCR). The technology was developed at Georgetown University in Washington, D.C. by Drs. Richard Schlegel and Xuefeng Liu with their team, first described in 2012 [38], and its detailed protocol was published in 2017 [39]. Deemed a “valuable” model for precision oncology [4], “high-quality laboratory model” [37], “new generation functional diagnostic” [40], and “exciting technological development” [12], the CCR technology became a “major breakthrough system that enables generation of the first inexhaustible patient-specific biobank of primary epithelial cells and cancer cells from the same patient” [41]. Shortly after its development, cell conditional reprogramming emerged as a powerful tool not only in cancer research, but also in other areas of biomedical science [42].

Conditional cell reprogramming is a very efficient cell culture method that allows immortalization of both normal and cancer cells in the laboratory without infecting or transfecting cells with any viral or cellular genes [38,39]. Therefore, the cells’ native genomic composition remains unchanged. Consequently, potential therapy can be tested on a patient’s own cancer cells (with a control of the patient’s own normal cells) [43]. Thus, the CCR method constitutes an important step toward personalized medicine. The conditionally reprogrammed (CR) cells are immortalized not by genetic manipulations, but by application of a cell culture medium containing Y-27632, a Rho-kinase (ROCK) inhibitor [44,45,46], and co-culture with irradiated Swiss-3T3-J2 murine fibroblasts (feeder cells) [47]. Induction of conditional cell reprogramming is very fast; it occurs on average within 2 days. Under CCR conditions, the cells proliferate indefinitely and do not differentiate as long as the conditions are maintained [39,48]. Conditional cell reprogramming is reversible; even following a long, multi-passage cell culture, after withdrawing the CCR culture conditions, cultured cells can be differentiated into the original tissue that they were obtained from [15,38,48]. Conditionally reprogrammed cancer cells form tumors when injected into adequate mouse model [15,38,49,50]. The cells are reprogrammed to the phenotype of adult stem cells [51]. They do not express high levels of embryonic stem cell markers, such as Sox-2, Oct-4, and Nanog, but they express an increased level of α6 and β1 integrins, ΔNp63α, CD44, and hTERT (human telomerase reverse transcriptase) [48]. They also show a decreased Notch signaling and an increased level of nuclear β-catenin [48,52].

Conventional methods used for immortalization of primary cells include introduction of viral genes (SV40 virus large T antigen, HPV E6/E7 oncoproteins), overexpression of hTERT and other genes, and induction of pluripotent stem cells (iPSc) [53,54,55]. In contrast to CCR technology, they require various genetic manipulations to achieve cell immortalization. Subsequently, genomic instability, loss of some genetic and biological properties of immortalized cells, and even tumorigenicity may be observed after the procedures. These methods are usually expensive and time-consuming. For each specific method, disadvantages such as loss of tissue heterogeneity in transformed cell lines or inapplicability of iPSc for a high-throughput drug screening assays are shown Table 1.

In contrast to conventional immortalization methods, CCR technology does not require introduction of exogenous genes, is fast, reversible, and inexpensive [39]. It has to be emphasized, however, that the technology has its weaknesses as well. It allows the growth of both malignant and non-malignant cells from original tissue, and these two cell populations are difficult to distinguish. Additionally, the growth of non-malignant cells is often preferentially promoted in the culture [56,57] and special methods need to be used to successfully isolate cancer cells [15,39,49]. Importantly, tumor-associated fibroblasts and stromal cells are inhibited in the co-culture [39], and conditionally reprogrammed cells do not always recapitulate all properties of differentiated cells of tissue of origin [58]. Moreover, Y-27632 ROCK inhibitor induces cytoskeleton remodeling; therefore, it may interfere with cell morphology and motility [44,59].

**Figure 1:**
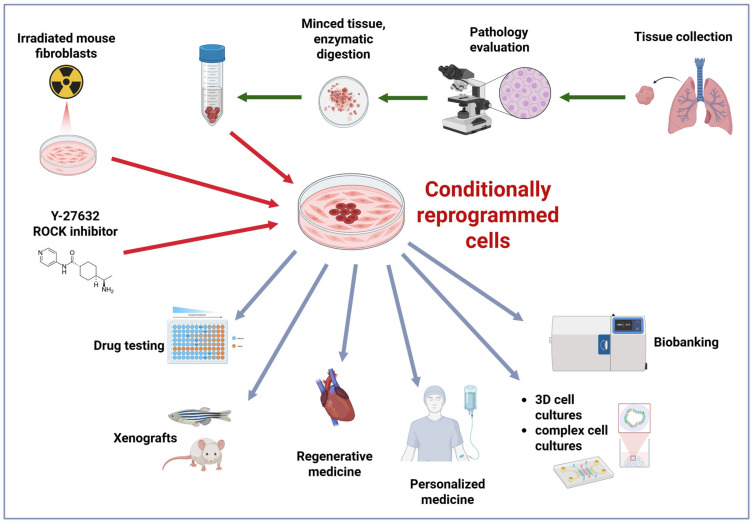
The outline of the clinical specimen collection and applications of the conditional cell reprogramming technology. Figure created using BioRender (https://app.biorender.com/, accessed on 1 August 2025).

The CCR technology has been shown to be applicable mainly to epithelial cells; however, it is possible to optimize cell culture conditions for other cell types [39,59,60]. Conditional cell reprogramming is a very effective and relatively non-expensive technique: within a few days, it is possible to obtain millions of fast-growing cells [39,43]. As shown in Figure 1 and described previously in detail [39], the procedure requires collection of patient tissue, including clinical biopsies [61], patient-derived xenografts (PDXs) [62,63], or liquid tissue samples, such as urine [64]. Clinical samples then undergo enzymatic/mechanic processing as necessary and are co-cultured with irradiated feeder cells in the CCR medium containing Y-27632 ROCK inhibitor. To avoid physical contact between cultured epithelial cells and feeder cells, conditioned medium (CM; medium incubated on irradiated feeder cell, collected and used as a cell culture medium [39]) is utilized. In some cases, the CCR method is optimized, i.e., modified depending on specific in vitro culture requirements of the particular cell line [39,59,60]. Generated cell lines can be cultivated in the laboratory for numerous passages without accumulation of chromosomal [15] or genetic changes [65], subsequently biobanked [15], and used for a variety of applications [42,66] (Figure 1). Conditional reprogramming is most commonly applied to generate human cells [39,43]; however, it has been utilized for animal (e.g., murine, canine, equine) cells as well [67,68,69].

The mechanisms of the conditional cell reprogramming are complex and involve multiple signaling pathways, such as TGF-β/SMAD, NOTCH, Rho/ROCK, and PI3K/AKT. Irradiated feeder cells and Y-27632 ROCK inhibitor stimulate cell proliferation by hTERT gene induction and increasing telomerase activity, as well as by cytoskeleton remodeling and deregulation of p16/Rb (retinoblastoma protein) pathway, respectively [38,42,45,66,70,71]. Importantly, the link between p53 pathway and hTERT activation regulates conditional cell reprogramming in epithelial cells [72]. Y-27632 inhibits apoptosis through Rho/ROCK-I/MLC pathway, immortalizes keratinocytes by cooperating with MYC [44], mitigates cell differentiation and growth arrest via Rho/ROCK and NOTCH pathways, and may prevent cell senescence by perturbing the p16/Rb-signaling pathway [38]. It is also involved in regulating cell differentiation by interference with the TGF-β/SMAD pathway [73]. Irradiated 3T3-J2 mouse fibroblasts activate EGFR, VEGFR, HGF, and HER2 receptors [74], subsequently affecting the STAT, MAPK, and PI3K/AKT signaling pathways [75]. They also stimulate the non-canonical pathway of β-catenin by increasing PP2Ac activity [52]. Moreover, irradiated feeders enable attachment of CR cells by producing extracellular matrix proteins, such as laminins, glycoproteins, and collagen [76].

The combination of both factors, Y-27632 and irradiated feeder cells, seems to be crucial for induction of proliferation and adult stem-like state of conditionally reprogrammed cells [48], as the co-culture with feeder cells is required for the cell immortalization induced by ROCK inhibitor [38,45]. In the absence of feeder cells, Y-27632 increases cell proliferation, but these cells cannot bypass senescence, so they cannot be propagated indefinitely. Feeder cell irradiation is necessary, since it stimulates apoptosis-correlated production of diffusible factors that contribute to cell immortalization [71,77]. The cell culture conditions can be modified, however. Physical contact between feeder cells and conditionally reprogrammed cells is not necessary; therefore, feeder-conditioned medium can be applied [77]. A plethora of secretory factors released by feeder cells are shown by Ligaba et al. These factors (and/or their combinations), including numerous phosphoproteins, can potentially be applied as a new generation of the CCR technique used as a preclinical disease model [71]. Moreover, Jeong and colleagues demonstrated, using three small molecules, Y-27632 (ROCK inhibitor), A83-01 (TGF-β inhibitor), and LDN193189 (BMP, bone morphogenetic protein inhibitor), enabling a long-term culture of salivary gland cells. However, their technique preferentially stimulated the growth of basal ductal progenitor cell population [78]. Crucially, the knockout of ROCK has not the same effect as using the ROCK inhibitor, Y-27632, as the conditionally reprogrammed cell culture. The knockout of a single isoform of ROCK, or even of both isoforms, ROCK1 and ROCK2, does not reprogram epithelial cells and does not promote cell culture propagation. Moreover, other known ROCK inhibitors cannot substitute for Y-27632. It is clear that Y-27632 acts beyond ROCK inhibition, as the drug affects other mechanisms and signaling pathways. Thus, the necessary conditions for conditional cell reprogramming require a collective activity of Y-27632 ROCK inhibitor and irradiated feeder cells [79,80].

**Figure 2:**
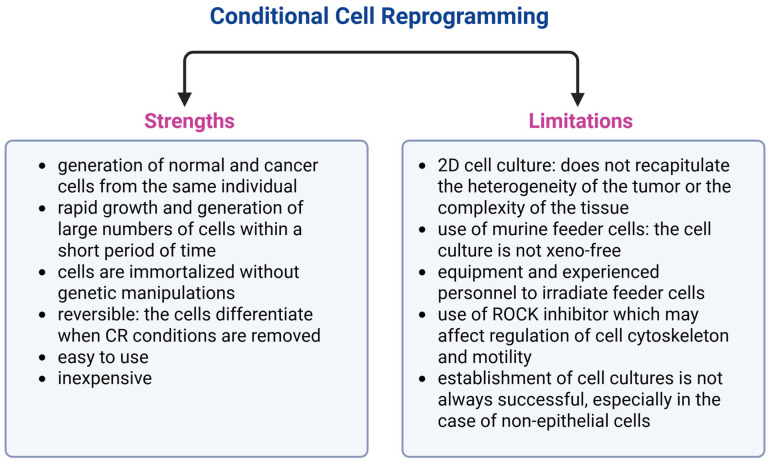
Major strengths and limitations of the conditional cell reprogramming technology. Figure created using BioRender (https://app.biorender.com/, accessed on 1 August 2025).

Despite its strengths, such as obtaining a large number of proliferating cells within a short period of time, conditional cell reprogramming technology does not completely recapitulate the heterogeneity of a tissue or tumor (Figure 2). Often, feeder cells inhibit the growth of mesenchymal cells, therefore limiting the use of the method as a reliable model of the interactions between tumor cells and stromal cells [39]. However, especially after optimization of cell culture conditions, CCR technique can preserve heterogenous phenotype of a cancer. This phenomenon was observed in conditionally reprogrammed neuroblastoma cells containing two distinct populations, adrenergic and mesenchymal, typical for neuroblastoma tumors in vivo [59]. Moreover, numerous studies demonstrated that the CR cells of various origins preserved their behavioral and transcriptome diversity that could reflect different risk profiles for human breast cancer [61]; CR tongue squamous cell carcinoma cells resembled the morphology and histologic characteristics of the tumor of origin after inoculation into immunodeficient mice [15]; and non-small cell lung cancers cells as well as breast cancer cells generated using CCR technology faithfully maintained the molecular characteristics of original tumors [65,81]. Strengths and weaknesses of conditional cell reprogramming are summarized in Figure 2.

There are studies showing that the ROCK inhibitor, Y-27632, can be applied to the conditionally reprogrammed cell culture conditions even for generating cell lines from cancers that are dependent on ROCK signaling. For example, the Rho/ROCK axis is important for the cytoskeleton regulation and formation of neurites in neuroblastoma cells, although it should be noted that their growth was slower in the culture conditions without Y-27632 [59,82,83]. Further studies, however, are advised for applying CCR technology for the research of malignancies that are Rho/ROCK-dependent.

## 3. Conditional Cell Reprogramming Applications in Cancer Research

The major strengths of the conditional cell reprogramming technology are the easy handling and the ability of culturing large numbers of immortalized, genetically stable cells within a short period of time [15]. The main weakness is that the cell culture setting is two-dimensional; thus, the model does not recapitulate the complex structure of tumor or normal tissue. Importantly, however, the CCR technology can be coupled with other, more complex and precise disease models, e.g., zebrafish in vivo model [84], in vitro 3D cell cultures [82] or PDXs [62], thus still proving a valuable step towards personalized medicine.

Conditionally reprogrammed cells may serve as a source of fast-growing cells that can facilitate and accelerate research using patient derived xenografts. For example, prostate cancer CR cells, in contrast to normal prostate cells, form tumors in SCID mice, thus providing a new functional platform to study prostate cancer [38,49]. Moreover, CCR technology can be used for establishing viable cell lines from PDX tumors (generated from human bladder, lung and ovarian cancers) without negatively affecting the original tissue genetic patterns and biological properties [62,63]. Interestingly, re-implanted CR-PDX cells retain histological and drug response characteristics of the parental PDX tumor [63]. These studies show that the CCR technology can be used to establish a reliable 2D cell culture model that complements in vivo PDX model.

Recently, the usage of CCR technology in cancer research has vastly increased. CR cells have been used in research on breast cancer [61,85,86,87,88,89], prostate cancer [49,58,90,91,92,93,94,95,96,97], head, neck and salivary gland cancers [15,50,98,99], various urological cancers [62,64,100,101,102,103,104], melanoma [105], cervical cancer [106], and lung and respiratory tract cancers [81,107,108,109,110,111], although it has to be noted that the attempts of using CCR technology for lung and pharyngeal cancer samples were not always successful [56,57,112]. Conditionally reprogrammed cells have also been used in digestive system oncology [113], including gastrointestinal cancer [114], colorectal cancer [115,116,117], pancreatic carcinoma [118,119,120], hepatocellular carcinoma, and other liver diseases [121,122,123]. That work demonstrates that CR cells are utilized as models for various cancer diseases, as a platform for drug testing and screening, as well as a valuable tool to study mechanisms of tumorigenesis. Besides cancer research, CR cultures are being used in other areas, such as virology [69,124,125,126,127,128,129,130], airway disorders and functions [131,132,133,134,135,136,137,138], asthma and cystic fibrosis [139,140], reproductive tract functions and diseases [141,142,143,144], kidney injury [145], regenerative medicine [146], studies on cell proliferation [46,47,52,71,147,148], and development of new diagnostic assays and therapies [149].

Conditionally reprogrammed cell models are valuable for commonly occurring cancers. Some excellent reviews on CCR technology in general, or focusing on particular cancer types, were published recently [42,64,85,100,104,150]. The current one specifically focuses on rare cancers and the significance of conditional reprogramming for this group of malignancies. As in vitro models are scarce and clinical samples are difficult to obtain in rare cancer research, new preclinical models are extremely important. Thus, the aim of this review is to present the conditional reprogramming technology in rare cancer research. Rare cancers presented in this review have different origins, develop in different organs, and have different genetic and phenotypic characteristics. Importantly, most of them are of non-epithelial origin, thus showing a new research area in which CCR technology, initially applied mostly to epithelial cells, can be utilized.

## 4. Conditionally Reprogrammed Cells as Preclinical Model for Rare Cancers

### 4.1. Neuroblastoma

Neuroblastoma is a rare cancer of autonomic nervous system that occurs mostly in children younger than 5 years. It is an extremely complex malignancy with numerous genetic and biological variations, various signaling pathways regulating pathogenesis, as well as clinical heterogeneity related to the severity of the disease and patterns of drug resistance, and poor survival prognosis. Due to the rarity and the complex nature of the malignancy, therapeutic options are still insufficient for patients’ long-term survival. Currently, chemotherapy, immunotherapy, radiotherapy, surgery, stem cell transplantations and combinational therapies are utilized. Moreover, advanced methods such as targeting exosome signaling via non-coding RNA, targeting cancer stem cells and epithelial-to-mesenchymal transition (EMT), various monoclonal antibodies, a combination of chemotherapeutic agents and developmental retinoids, as well as numerous types of drug delivery strategies constitute new promising therapeutic options [151]. Hence, there is an urgent need for the development of preclinical neuroblastoma models facilitating preclinical evaluations of potential therapies, especially for therapies effective against disease initiation and metastasis [152,153,154].

**Figure 3:**
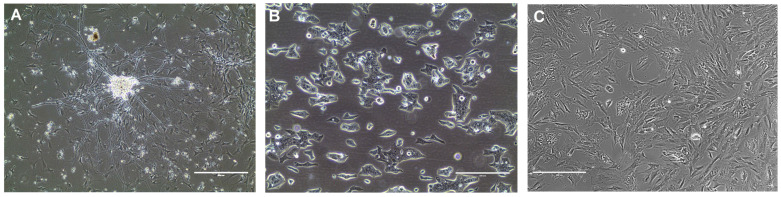
Cell lines obtained from tissues collected from rare cancers and generated using conditional reprogramming technology: (**A**) murine neuroblastoma cell line; (**B**) neuroendocrine cervical cancer cell line; (**C**) GIST cell line. Scale bars: 400 µm (**A**,**C**) and 200 µm (**B**).

Murine neuroblastoma cell lines that were generated using CCR technology were described in 2020 for the first time ([59], Figure 3A). Cell culture settings were an optimized conditional reprogramming method: cells were grown in the CR medium supplemented with Y-27632, without feeder cells, at 2% O_2_ (Table 2). Twenty-one cell lines were obtained from tumors growing in a commonly utilized model of human neuroblastoma, TH-MYCN transgenic mice. All of the cell lines retained typical heterogenous phenotype with two distinctive cell populations: mesenchymal and adrenergic, and they were positive for specific biomarkers of neuroblastoma lineages’, as well as for neuronal markers. Their tumorigenic phenotype was confirmed using migration, invasion, and anchorage-independent growth assays in vitro. Passaged for numerous passages and biobanked, neuroblastoma CR cells were subsequently utilized to discover the role of neuropeptide Y receptor in neuroblastoma cell motility [83], as well as tested for their ability to grow in 3D cell culture settings [82]. The main disadvantage of this neuroblastoma in vitro model is that these cells are not human. However, the plans to obtain and culture neuroblastoma cells collected from patients have been made.

Neuroblastoma is an extremely complex malignancy; therefore, its therapy must be carefully designed and personalized, depending on the disease severity and stage. CCR technology, as a personalized preclinical model, may be useful for screening potential neuroblastoma treatments. It may be particularly successful in testing the therapies that affect EMT, as the CCR technology preserves the heterogenous phenotype of the disease (Figure 3A). Other current approaches in applying preclinical neuroblastoma models for the development of new therapies include identification of residual malignant persister cells in tumor samples using single-nucleus RNA sequencing and bulk whole-genome sequencing [162,163], multiomic detection of cellular and molecular interactions of cells within the neuroblastoma tumor for potential use of immunotherapies [164,165], as well as 3D in vitro models for neuroblastoma microenvironment studies [166].

### 4.2. Neuroendocrine Cervical Carcinoma

Neuroendocrine cervical cancer is a highly aggressive malignancy. Because of its rare occurrence there are no relevant preclinical models of this disease. Using CCR technology, one unique cell line was generated from aggressive large cell neuroendocrine cervical carcinoma metastatic to liver [60]. The cancer tissue was collected from a 27-year-old patient who passed away 3 months later. The cells grew in 3D spheres initially, then formed a rapidly proliferating monolayer (Figure 3B). They required a modified CR cell culture condition: without feeders but collagen-coated cell culture vessel for their growth (Table 2). Genome of human papillomavirus type 16 was found to be integrated into the cellular genome, adjacent to the *Myc* gene. However, a 40-fold amplification and overexpression of *Myc* was the driver of transformation of these cells, rather than the HPV-16 oncogene expression. Interestingly, the generated cell line harbored a p53 mutation, which is uncommon for most cervical cancers. Its transformed phenotype was shown in vitro (invasion and migration assays, anchorage-independent growth in soft agar) and in vivo by tumor formation in immunodeficient mice. One possible explanation is that p53 may have different activities in cervical neuroendocrine cells and in cervical squamous cells. However, the establishment of a reliable cancer model has not been fully achieved, since the paper refers to one study and a single, very unique, cell line only.

### 4.3. Neuroendocrine Prostate Cancer

In contrast to prostate cancer in general, neuroendocrine prostate cancer is very rare. It is an extremely aggressive disease, with its mechanisms not very well known because of lack of adequate models in vitro [167]. Ci et al. [160] described a series of experiments combining xenografts and CCR technology. Tumor specimens were collected from prostate cancer patients and transplanted into immunodeficient mice. After numerous in vivo passages, prostate adenocarcinoma PDX tumors were harvested and grown using standard CR conditions (Table 2). Notably, it was observed that CR conditions partially mimicked castration in vivo: prostate adenocarcinoma PDX transdifferentiated to prostate neuroendocrine cancer after castration of a mouse host and subsequent tumor regression. When CR-PDX cells were re-transplanted to mice, the resultant tumors appear to be neuroendocrine prostate cancers, different from parental adenocarcinoma tumors. Histological and transcriptomic analyses demonstrated that the cells lost their adenocarcinoma markers, and expressed neuroendocrine ones. Also, neuroendocrine genes were enriched, in contrast to androgen receptor signaling genes. Cell culture conditions used in this study were a combination of CR conditions and PDX samples and, interestingly, a transdifferentiation process occurred in the course of the study. Therefore, more studies aiming at the generating and characterizations of this type of cancer are necessary to claim an establishment of a new fully reliable preclinical cancer model.

### 4.4. GIST

Gastrointestinal stromal tumors (GIST) are rare abdominal tumors. Histologically, most of them consist of spindle-shaped, mesenchymal cells [168]. Clinical specimens of the GIST were collected from a patient at the Georgetown University hospital. The tumors were processed and cultured using conditional reprogramming technology (Figure 3C). Cell cultures confirmed that the proliferation and growth of fibroblast/stromal cells is inhibited by J2 feeder cells, as observed before [47,169]. Thus, the cell culture method was optimized for GIST cells [39] (Table 2). One conditionally reprogrammed cell line was generated and biobanked. A long-term growth of the cell line and reproducibility was not assessed; moreover, the single cell line generated was not characterized. The data are promising, but more are necessary for the establishment of the CCR technology-based in vitro model for GIST.

### 4.5. Gliomas and Other Glioneural Tumors

#### 4.5.1. Ependymoma

Pediatric spinal ependymomas are uncommon cancers of the central nervous system. Because of their rarity, there are no sufficient preclinical models available [170]. A novel preclinical ependymoma model was described in 2017 [155]. The tumor sample was collected from a 12-year-old patient with spinal myxopapillary ependymoma. The cells were grown in optimized conditional reprogramming cell culture (Table 2). Importantly, in comparison to their controls, CR ependymoma cells demonstrated elevated expression of genes encoding High Mobility Group Protein AT-hook 1 (HMGA1), High Mobility Group Protein AT-hook 2 (HMGA2), cMYC, HOXB13, and HOXA10 proteins.

In the second study, two other myxopapillary ependymomas collected from patients above 40 years old were cultured using CCR technology [156]. They were grown in modified CR conditions: CR medium mixed with conditioned medium (CM; CR medium conditioned on irradiated J2 feeder cells [39]) (Table 2). The cell culture method was slightly modified when compared to the one used by the same team before; however, it still did not fully support the growth of CR ependymoma cells. Further optimization of the CCR technology for the establishment of ependymoma in vitro models is necessary.

#### 4.5.2. Pilocytic Astrocytoma

Pilocytic astrocytoma cell line was generated using optimized CCR technology (Table 2) [156]. The tumor was collected from a 14-year-old patient with neurofibromatosis type 1. Conditionally reprogrammed cells were growing rapidly, were genetically stable for numerous passages, and positive for vimentin and neural progenitor markers, nestin, and NG2. These cultured cells were used for drug testing in vitro, as well as characterized in various biological assays. Reduced *NF1* expression was confirmed on the mRNA level. *BRAF* p.V600E mutation was detected by pyrosequencing and PCR analysis. The cells formed tumors in immunodeficient mice, and the patient’s glial cells were observed in the brain and leptomeninges of these mice. In zebrafish xenografts, the cells were migrating along developing zebrafish spinal cord. Senescence induction was evaluated using β-galactosidase staining, and cell cycle proteins (p21, p27) were detected by Western blot analysis. Additionally, two other pilocytic astrocytoma CR cell lines were grown; however, the growth was not as abundant. The results suggest that the studies on the application of conditional reprogramming for viable, fast-growing, and reliable astrocytoma cell lines are needed.

#### 4.5.3. Other Gliomas

The same team was able to grow other gliomas and glioneural tumors using optimized conditional cell reprogramming technology (Table 2). Among them were anaplastic pleomorphic xanthoastrocytoma (this CR cell line forms orthotopic tumors in nude mice); anaplastic gliomas grade III and III/IV; gangliogliomas grade I and III; low grade neuroepithelial tumors; and low-grade gliomas with pilomyxoid and ependymal features [156]. That extensive study confirms that CR technology seems to be a promising method to be used as a preclinical model for drug-screening and mechanistic assays, especially in cases of rare tumors that are difficult to propagate in standard media.

### 4.6. Ameloblastoma

Six conditionally reprogrammed cells lines from excised ameloblastomas (odontogenic tumors) were generated [145]. They were grown in modified CR cell culture conditions (Table 2) for six to eight passages only. Afterwards, to avoid cellular senescence, cell lines were immortalized with hTERT transduction at early passage. The cell lines expressed amelotin, ameloblast-associated gene, and harbored various ameloblastoma driver mutations in MAPK (mitogen-activated protein kinase) pathway, e.g., KRAS, NRAS, BRAF, FGFR2), and Hedgehog pathway (SMO). Two of them were the first tumor cell lines established that carried SMO mutations. Generated cells were used in drug sensitivity assays and oncogene dependency assays. However, because human ameloblastoma cell lines grew in CR conditions only for a limited number of passages, it has to be emphasized that in this case, a reliable disease model was not established.

Before human ameloblastoma cell lines, the same team successfully generated cells from an analog of human ameloblastoma, namely canine acanthomatous ameloblastoma [158]. Canine ameloblastoma CR cell lines were immortalized in modified CR cell culture conditions (Table 2) and were tested for their sensitivity to drugs. This study shows the potential of establishing CCR technology-dependent in vitro models that can be applicable in veterinary medicine.

### 4.7. Laryngeal and Hypopharyngeal Carcinoma

Laryngeal and hypopharyngeal carcinoma is a rare type of head-and-neck cancer. Its prognosis is poor due to inefficient treatment; therefore, the development of new potential therapies, including chemotherapies, is of utmost importance [171]. Dong et al. [161] describe establishment of cell lines from patients with a rare type of namely laryngeal and hypopharyngeal squamous cell carcinomas (LHSCC). They generated 28 cell lines (both tumor and corresponding normal control) from 50 clinical specimens, using standard CCR technology (Table 2). The cells were genetically stable, recapitulated genetic changes typical for LHSCCs, and expressed an epithelial cell marker (pan-keratin), as well as a head-and-neck cancer biomarker, CD44 (CD44 expression levels were higher in tumor cell lines, in comparison to normal cells). Generated CR cell lines’ tumorigenicity was evaluated by their potential to form tumors in nude mice. Tumor cells injection resulted in formation of tumors, while normal CR cells did not show that effect. CR LHSCC cells, growing in 2D and 3D settings, as well as xenografts, were used for various drug and radiation sensitivity testing. The authors of the study claim that even though the successful growth rate of the CR cell lines was not the highest, it was still superior to xenograft and organoid 3D growth. Moreover, conditional reprogramming alone and in combination with other preclinical models may be useful for identification of new potential drug targets, as well as for discovery of new therapeutic agents for the laryngeal and hypopharyngeal carcinoma.

### 4.8. Adenoid Cystic Carcinoma

Adenoid cystic carcinomas are slow-growing but highly lethal tumors, neuroinvasive, recurrent, and often metastatic. The treatment is limited due to the elusive molecular nature of the tumor, as well as the lack of adequate in vitro screening models. Establishment of six ACC conditionally reprogrammed cell lines (1 from primary tumor and 5 from xenografts) has been described [159]. The cells were grown in optimized CR cell culture conditions (Table 2). In contrast to normal salivary gland cells, they expressed ACC markers previously introduced to validate stem cell identity of cultured cells (*SOX10*, *NOTCH1*, *FABP7*, *NTRK3/TrkC* and *PROM1/CD133*), and formed spheroids in 3D cultures and tumors in immunodeficient mice, proving to be a useful source for adenoid cystic carcinoma samples for personalized oncology.

Additionally, CR adenoid cystic carcinoma cells collected from xenografts were injected into the zebrafish model, thus establishing a new approach for the preclinical modeling of this malignancy [84]. Two conditionally reprogrammed ACC xenograft cell lines were established, using optimized CCR technology (Table 2). The cells maintained their transformed potential, as shown in in vitro soft agar and invasion assays. They were grown for a limited number of passages and subsequently injected into zebrafish embryos. Conditionally reprogrammed cells, PDXs, and zebrafish models were used for drug sensitivity testing, and the results were similar. The authors concluded that these cultures can be useful as models for basic and translational studies.

## 5. Conclusions

Preclinical in vitro disease models are indispensable in every area of cancer research. Conditional cell reprogramming is a novel technology that can be used as a 2D in vitro model for cancer and other diseases. Obtaining fast-growing non-differentiated cells that can be reversibly immortalized with cell culture conditions only without any genetic modifications is the strength of this technology. Moreover, both normal and cancer cells collected from the same patients can be grown alongside each other, even in the case of rare cancers. Conditionally reprogrammed cells can also be generated from different stages of tumors. Furthermore, CR cultures preserve tissue heterogeneity to some extent; therefore, they may be applied to studies of cellular interactions in various tumors. However, it needs to be emphasized that CCR technology is still a two-dimensional cell culture system and suffers from the major weaknesses of that kind of setting, including poor representation of tumor/tissue complexity and the lack of adequate representation of the three-dimensional tissue structures (Figure 2). Additionally, attempts to establish viable cell lines without the optimization of the cell culture conditions are not always successful. Notably, in many cases of rare cancers, especially of non-epithelial origin (gliomas, neuroblastoma, neuroendocrine cervical cancer, ameloblastoma) the CCR technology needs to be modified and optimized (Table 2). Consequently, these studies confirm the decades-old discovery that irradiated 3T3-J2 cells inhibit the growth of tissue stromal cells. The necessary modification in most cases was the removal of the feeders from the cell co-culture.

Importantly, conditional cell reprogramming technique cultures can be combined with other, low-throughput, but more complex and precise platforms such as patient-derived xenografts and/or 3D systems (as used for culturing astrocytoma, adenoid cystic carcinoma, laryngeal and hypopharyngeal carcinoma). It suggests that these different types of preclinical models can work in synergy to advance cancer research. They can be utilized in numerous research areas, from evaluation of the malignancy mechanisms and identification of potential therapeutic targets, to high-throughput screening of potential anticancer drugs and the determination of mechanisms of cellular susceptibility to these agents.

The CCR technology (and its combinations with other methods) may be even more important in often neglected rare cancer research area, due to their elusiveness, unique tumorigenicity mechanisms, and infrequent occurrence. The main challenges of the burden of rare cancers are insufficient patient numbers and therefore difficulty in establishment of clinical trials for potential drugs, and, consequently, inadequate numbers of effective therapies and poor disease prognosis for patients [33,36]. There is a notable lack of relevant and reliable models to study these malignancies. Thus, the development of new preclinical models is crucial. This article reviews the conditional cell reprogramming technology as a novel and useful model, demonstrating its strengths and limitations, as well as applications for various rare cancers studies. Alone and in combination with other technologies, it can be used in various aspects of basic and translational cancer research related to rare diseases.

## Figures and Tables

**Table 1:** Comparison between selected methods of cell immortalization in vitro.

	Transformed Cell Lines	iPSc *	CR Cells **
Success rate	Medium	Medium	High
Lifespan	Long	Long	Long
Timing	1–2 months	2–10 weeks	1–10 days
Expansion	High	Medium	High
Genetic stability	Low	Medium	High
Tissue specificity	Low	Low	High
Heterogeneity	No	Medium	Medium
Differentiation	Low	Medium	High
High-throughput drug screening applicability	High	Low	High
Low-throughput drug screening applicability	High	High	High
Cost	Low	Medium	Low

* iPSc: induced pluripotent stem cells; ** CR cells: conditionally reprogrammed cells.

**Table 2:** The list of conditionally reprogrammed cell lines generated from rare cancers. Either standard (CR medium + Y-27632 + feeder cells) or modified (CM conditioned medium, ACC adenoid cystic carcinoma medium, low O_2_ level, collagen coating) CR technology was utilized.

Tumor		Standard CR Technology			CM Medium	ACC ** Medium	2% O_2_	Collagen Coating	Number of Generated CR Cell Lines	Refs
		CR Medium	Feeder Cells	Y-27632						
Neuroblastoma (murine)		+		+			+		21	[59,82,83]
Neuroendocrine cervical cancer		+		+				+	1	[60]
Ependymoma		+		+			+		1	[155]
		+		+	+				2	[156]
Pilocytic astrocytoma		+		+	+				3	[156]
Pleomorphic xanthoastrocytoma		+		+	+				1	[156]
Other lower-grade gliomas		+		+	+				8 (total)	[156]
GIST		+		+			+		1	[39]
Ameloblastoma	human *	+		+	+				6	[157]
	canine	+		+	+				4	[158]
ACC	[159]	+		+	+				6 (total)	[159]
	[84]	+	+	+		+			2	[84]
Neuroendocrine prostate cancer		+	+	+					1	[160]
Laryngeal and hypopharyngeal carcinoma		+	+	+					28	[161]

GIST: gastrointestinal stromal tumor; ACC: adenoid cystic carcinoma; CR: conditional reprogramming; CM: conditioned medium. ** ACC medium consists of CR medium supplemented with noggin, SB202190, rhFGF, CHIR-99021 and Wnt-3a [84]. * Human ameloblastoma cell lines grew for 6-8 passages only in CR conditions.
